# Estrogen stimulates fetal vascular endothelial growth factor expression and microvascularization

**DOI:** 10.1530/JOE-23-0364

**Published:** 2024-06-07

**Authors:** Graham W Aberdeen, Jeffery S Babischkin, Gerald J Pepe, Eugene D Albrecht

**Affiliations:** 1 Departments of Obstetrics, Gynecology, Reproductive Sciences and Physiology, University of Maryland School of Medicine, Baltimore, Maryland, USA; 2 Department of Physiological Sciences, Eastern Virginia Medical School, Norfolk, Virginia, USA

**Keywords:** estrogen, fetus insulin sensitivity programming progeny, microvascularization, VEGF introduction

## Abstract

We recently showed that the ratio of capillaries to myofibers in skeletal muscle, which accounts for 80% of insulin-directed glucose uptake and metabolism, was reduced in baboon fetuses in which estrogen was suppressed by maternal letrozole administration. Since vascular endothelial growth factor (VEGF) promotes angiogenesis, the present study determined the impact of estrogen deprivation on fetal skeletal muscle VEGF expression, capillary development, and long-term vascular and metabolic function in 4- to 8-year-old adult offspring. Maternal baboons were untreated or treated with letrozole or letrozole plus estradiol on days 100–164 of gestation (term = 184 days). Skeletal muscle VEGF protein expression was suppressed by 45% (*P* < 0.05) and correlated (*P* = 0.01) with a 47% reduction (*P* < 0.05) in the number of capillaries per myofiber area in fetuses of baboons in which serum estradiol levels were suppressed 95% (*P* < 0.01) by letrozole administration. The reduction in fetal skeletal muscle microvascularization was associated with a 52% decline (*P* = 0.02) in acetylcholine-induced brachial artery dilation and a 23% increase (*P* = 0.01) in mean arterial blood pressure in adult progeny of letrozole-treated baboons, which was restored to normal by letrozole plus estradiol. The present study indicates that estrogen upregulates skeletal muscle VEGF expression and systemic microvessel development within the fetus as an essential programming event critical for ontogenesis of systemic vascular function and insulin sensitivity/glucose homeostasis after birth in primate offspring.

## Introduction

There is a prevalent increase in the incidence of type 2 diabetes mellitus (T2DM), particularly in the younger generation, in the USA (Cowie *et al.* 2018, Lin *et al.* 2020). However, little is known about the cause of this disturbing trend. The hormonal milieu of pregnancy has an extremely important role in programming the developmental events in the fetus that control metabolic function after birth in the offspring (Hoffman *et al.* 2021). Thus, circumstances in which the levels or action of estrogen are curtailed or reduced during human pregnancy, e.g. preterm birth, aromatase gene mutation, or endocrine disruptors that interfere with estrogen receptor action, lead to T2DM in offspring (Hofman *et al.* 2004, Darendeliler *et al.* 2008, Fukami *et al.* 2009, Alonso-Magdalena *et al.* 2010, Paz *et al.* 2017). We have recently shown that offspring born to baboon mothers, in which the serum levels of estradiol were suppressed by maternal administration of the aromatase inhibitor letrozole during the second half of pregnancy, exhibited insulin resistance and glucose intolerance. These effects were prevented by maternal administration of letrozole plus estradiol (Maniu *et al.* 2016, Pepe *et al.* 2016). Adult baboon offspring delivered to letrozole-treated/estrogen-deprived mothers eventually developed a deficit in first-phase pancreatic insulin release (Pepe *et al.* 2016), a process that leads to T2DM (Del Prato & Tienga 2001). Although the mechanisms that underpin the development of insulin resistance in estrogen-suppressed baboon offspring are unknown, insulin receptor signaling and glucose transporter protein levels were unchanged in the skeletal muscle of baboon fetuses deprived of estrogen (Maniu *et al.* 2016, Kim *et al.* 2017).

The delivery of insulin and glucose to target cells is facilitated by the terminal arterioles and capillaries (Clark *et al.* 2002, Chiu *et al.* 2008, Richards *et al.* 2010, Horton & Barrett 2021), microvessels that are present in very high numbers in skeletal muscle (Kubínová *et al.* 2001, Egginton 2009, Glancy *et al.* 2014, Williams *et al.* 2020). Consequently, skeletal muscle accounts for over 80% of insulin-directed glucose uptake and metabolism (De Fronzo *et al.* 1985, Lillioja *et al.* 1987, Wolfe 2006). Although the regulation of the microvascular network has been relatively well established in adults (Prior *et al.* 2014, Keske *et al.* 2017, Sangha *et al.* 2021), little is known about the regulation of microvessel development in the fetus and its impact on insulin sensitivity after birth (Thompson & Regnault 2011, Clough 2015). We have recently shown that the ratio of the number of capillaries per number of microfibers in skeletal muscle was reduced in fetuses of letrozole-treated/estrogen-deprived maternal baboons (Albrecht *et al.* 2022). However, microfiber size, but not number, was also decreased in fetuses of letrozole-treated baboons (Kim *et al.* 2022). Therefore, in the present study, the number of capillaries was quantified and expressed per myofiber area to determine if it resulted in a change with estrogen deprivation consistent with that expressed per individual myofiber, and thus may impact responsivity of skeletal muscle to insulin.

We also recently showed that brachial artery flow-mediated dilation (FMD), which reflects endothelial-dependent nitric oxide-mediated vasodilation (Corretti *et al.* 2002, Linke *et al.* 2005, Pyke & Tschakovsky 2005, Kooijman *et al.* 2008), was reduced in offspring of letrozole-treated baboons (Albrecht *et al.* 2022). Acetylcholine-induced arterial flow reflects endothelial-dependent nitric oxide-mediated vasodilation and prostaglandin-mediated vasocontractility (Boulanger *et al.* 1994, Kellogg *et al.* 2005, Medow *et al.* 2008, Stiegler *et al.* 2021). Therefore, to determine the impact collectively of nitric oxide- and prostaglandin-modulated responses, in the present study, brachial artery flow was quantified during the administration of acetylcholine to offspring delivered from untreated and estrogen-deprived baboons.

The factors that mediate the impact of estrogen on fetal microvascular development are unknown. Vascular endothelial growth factor (VEGF) has a well-established role in promoting angiogenesis in the adult, i.e. the formation and proliferation of capillaries (Ferrara 1999, Dvorak 2000, Wagner 2011), and estrogen stimulates VEGF expression in many estrogen receptor-positive tissues in adults (Cullinan-Bove & Koos 1993, Nayak & Brenner 2002, Higgins *et al.* 2006, Aberdeen *et al.* 2008). Estrogen receptor alpha and beta are expressed in capillary endothelial cells of skeletal muscle (Wiik *et al.* 2005, 2009), and estrogen promotes insulin sensitivity and glucose tolerance in skeletal muscle in adults (Deroo & Korach 2006, Gao *et al.* 2006, Belgorosky *et al.* 2009, Gavin *et al.* 2009, Riant *et al.* 2009). However, it remains to be determined whether the elevation of estrogen in primate pregnancy upregulates VEGF expression within the skeletal muscle of the fetus and thus affects the development of the fetal and offspring vascular network required for the onset of insulin sensitivity and glucose homeostasis after birth. Therefore, the present study builds on our recent study to determine the effect of *in utero* estrogen deprivation and restoration on expression of VEGF and myofiber capillary density in the skeletal muscle of the near-term baboon fetuses and the long-term impact on acetylcholine-induced vasodilation after birth in adult offspring.

## Materials and methods

### Animals

Female baboons *(Papio anubis)* were housed in rooms with a 12 h light:12 h dark cycle, received primate chow (Teklad Primate Diet, 2050, Envigo, Frederick, MD, USA), and fresh fruit twice daily. They had access to water *ad libitum* and were paired with male baboons during the ovulatory stage of the reproductive cycle, as estimated by the pattern of external sex skin turgescence. Day 1 of pregnancy was designated as the day preceding perineal deturgescence. Baboons were used in accordance with the U.S. Department of Agriculture regulations and the National Institutes of Health *Guide for the Care and Use of Laboratory Animals* (8th ed.). The experimental protocol was approved by the Institutional Animal Care and Use Committees of the University of Maryland School of Medicine and Eastern Virginia Medical School.

Pregnant baboons were randomly assigned as untreated, treated with aromatase inhibitor letrozole (4,4-[1,2,3-triazol-1-yl-methylene]bis-benzonitrate, Novartis Pharma AG, Basel, Switzerland; 115 µg/kg body weight/day, maternal s.c. injection) between days 100 and 164 of gestation (term = 184 days), or treated with letrozole (115 µg/kg body weight/day) plus estradiol benzoate (25 µg/kg on day 100 and increasing to 115 µg/kg body weight between days 120 and 164) to replicate the normal increase in estradiol.

Some of the fetuses were delivered via cesarean section on day 165 of gestation during isoflurane anesthesia. Two mL of blood samples were obtained from a maternal saphenous vein and umbilical artery for estradiol quantification. The fetuses were euthanized by i.v. injection of pentobarbital (100 mg/kg body weight). A section (5 mm^3^) of vastus lateralis was fixed in formalin and embedded in paraffin for quantification of VEGF protein by proximity ligation assay and capillary density by image analysis. The remaining fetuses were delivered spontaneously, and newborns were nursed by their mothers for 8 months. They were then weaned and fed standard primate chow (Teklad Primate Diet, 2050, Envigo) and fresh fruit twice daily, and water *ad libitum*. At 4–8 years of postnatal age (female baboons attain puberty at 3.5 years and male baboons at 4 years of age), acetylcholine-induced brachial artery dilation and blood pressure were measured after light anesthetization with propofol/ketamine supplemented with oxygen (1 L/min to maintain SpO_2_ greater than 95%). Approximately 1 week later, a glucose tolerance test was performed during propofol/ketamine anesthetization.

### Proximity ligation assay of VEGF

VEGF protein levels were quantified by proximity ligation assay, as described previously (Babischkin *et al.* 2019), in the skeletal muscle of fetuses obtained from seven untreated (five female, two male fetuses), six letrozole-treated (four female, two male fetuses), and five letrozole plus estradiol-treated (three female, two male fetuses) baboons. Briefly, paraffin-embedded transverse vastus lateralis sections (5 µm) were incubated with a rabbit polyclonal primary VEGF antibody (1:250 dilution, Thermo Fisher), followed by proximity ligation assay probes consisting of two secondary anti-rabbit antibodies, both against the lone primary antibody, but one with the PLUS oligonucleotide tag and one with the MINUS oligonucleotide tag. A ligation solution (supplied in the proximity ligation assay kit, Duolink MilliporeSigma), consisting of two oligonucleotide linkers complementary to each proximity ligation assay probe, was added. Tissue sections were then incubated with an amplification solution consisting of fluorescently labeled oligonucleotides and polymerase. The polymerization step employs rolling circle DNA amplification to generate a concatemeric oligonucleotide product linked to the antibody complex. The fluorescently labeled oligonucleotides hybridize to the rolling circle amplification product, with the resultant signal visible as a fluorescent dot. Tissue sections were then incubated with a mouse monoclonal antibody to Myosin Fast (1:250 dilution, Millipore Sigma) for myofiber identification and localization of VEGF, Alexa Fluor 488 donkey anti-mouse secondary antibody (Invitrogen/Thermo Fisher Scientific), and DAPI for detection of nuclei. The number of VEGF protein red proximity ligation assay signals was quantified by fluorescence microscopy and MetaMorph software (version 7.8.0.0; Molecular Devices, San Jose, CA, USA) and expressed per µm^2^ × 10^4^ areas of groups of skeletal muscle fibers. Negative controls included the substitution of normal rabbit serum for the primary antibody or the omission of one ligation probe.

### Skeletal muscle capillary density

Capillary density was quantified in the skeletal muscle of fetuses obtained from eight (six female, two male fetuses) untreated (i.e. six in which capillary/myofiber ratio was previously reported (Albrecht *et al.* 2022) and two additional animals), five (three female, two male fetuses) letrozole-treated (i.e. four previous (Albrecht *et al.* 2022) and one additional), and five (three female, two male fetuses) letrozole plus estradiol-treated (i.e. four previous (Albrecht *et al.* 2022) and one additional) baboons. Transverse sections (5 µm) of paraffin-embedded vastus lateralis tissue were labeled with a primary rabbit antibody to endothelial-specific von Willebrand factor (VWF, 1:250 dilution, Agilent Dako) and a mouse monoclonal antibody to Myosin Fast (1:250 dilution; MilliporeSigma) for capillary and myofiber identification, respectively. Tissue sections were incubated with a bridge biotinylated goat anti-rabbit secondary antibody (Vector Laboratories), followed by incubation with streptavidin Alexa Fluor 488 conjugate, Alexa Fluor 594 donkey anti-mouse secondary antibody (Invitrogen/Thermo Fisher Scientific), and DAPI for detection of the nuclei. Stained muscle sections were visualized via fluorescence microscopy and analyzed using analysis software (IP Lab). The number of VWF- positive endothelial cells was quantified on capillaries based on the size of the vessels (approximately 8 µm diameter), and results were expressed as the number per mm^2^ muscle fiber area using MetaMorph software in ten randomly selected skeletal muscle areas per section. A minimum of 550 myofibers were analyzed per section. Negative controls for fluorescent immunohistochemistry included the substitution of normal rabbit serum (Invitrogen/Thermo Fisher Scientific) for the primary antibody.

### Acetylcholine-induced arterial dilation and blood pressure

Acetylcholine-induced arterial dilation was quantified in offspring delivered from 11 (eight female, three male offspring) untreated (i.e. eight in which FMD was previously measured (Albrecht *et al.* 2022) and three additional animals), eight (three female, five male offspring) letrozole-treated (i.e. five previous (Albrecht *et al.* 2022) and three additional), and seven (three female, four male offspring) letrozole plus estradiol-treated (i.e. five previous (Albrecht *et al.* 2022) and two additional) baboons. Brachial artery dilation was quantified via an Acuson Sequoia 512 ultrasound (15L8 linear transducer, Siemens) on offspring at diastole before (basal level) and during a 5 min (eight values obtained at 30 s intervals) i.v. infusion of acetylcholine (8 µg/kg body weight) in baboons lightly anesthetized with i.v. propofol/ketamine.

Blood pressure was measured via a Dinamap Pro 400 V2 (GE Medical Systems) immediately preceding acetylcholine-induced arterial dilation in offspring obtained from 15 (nine female, six male offspring) untreated (12 in which FMD was previously reported (Albrecht *et al.* 2022) and three additional animals), 12 (seven female, five male offspring) letrozole-treated (ten previous (Albrecht *et al.* 2022) and two additional), and ten (five female, five male offspring) letrozole plus estradiol-treated (eight previous (Albrecht *et al.* 2022) and two additional) baboons.

### Glucose tolerance test

An i.v. glucose tolerance test was performed on baboon offspring according to the established method of Overkamp *et al.* (1997) in offspring delivered from 16 (eight female, eight male offspring) untreated (i.e. four of the offspring in which blood pressure or acetylcholine-induced arterial pressure was measured in the current study and 12 offspring in which a glucose tolerance was previously performed (Albrecht *et al.* 2022)), 18 (ten female, eight male offspring) letrozole-treated (i.e. ten of the offspring in which blood pressure or acetylcholine-induced arterial pressure measured in the current study and eight previous (Albrecht *et al.* 2022)), and 15 (seven female, eight male offspring) letrozole plus estradiol-treated (seven of the offspring in which blood pressure or acetylcholine-induced blood pressure measured in the current study and eight previous (Albrecht *et al.* 2022)) baboons. Baboons were fasted overnight, initially sedated with ketamine HCl (5–10 mg/kg body weight, i.m.), then lightly anesthetized with i.v. propofol/ketamine supplemented with oxygen (1 L/min). A bolus solution of dextrose (i.e. d-glucose, 0.25 gm/kg body weight) was injected into an antecubital vein at time 0, and blood samples (2.5 mL each) were obtained from a peripheral saphenous vein at –2 (i.e. basal), 1, 3, 5, 10, 20, 40, 60, and 90 min before/after dextrose administration.

Blood glucose levels were quantified via an iStat Portable Clinical Analyzer (Model #210003, Abbott Laboratories), and plasma insulin levels were quantified using a Siemens ADVIAR Centaur CP Immunoassay System (Siemens Healthcare). The insulin assay employed a monoclonal murine anti-insulin antibody and displayed a sensitivity of 2 µIU/mL, with intra- and inter-assay coefficients of variation of 5.7% and 5.9%, respectively, and exhibited no cross-reactivity with other peptides.

### Serum estradiol

Serum estradiol levels were quantified via an automated chemiluminescent immunoassay system (Immulite; Diagnostic Products).

### Statistical analysis

Serum estradiol levels were analyzed by one-way ANOVA with *post hoc* comparison of the means by Newman–Keuls multiple comparison test (GraphPad software). VEGF levels, capillary density, blood pressure, and glucose and insulin levels were analyzed by one-way ANOVA followed by the Tukey–Kramer multiple comparison test. Brachial artery dilation values were analyzed by Kruskal–Wallis ANOVA and Dunn’s multiple comparison test. The impact of the sex of the offspring on glucose and insulin levels and the HOMA was analyzed by a two-way ANOVA with the treatment group and sex as the main effects.

## Results

### Serum hormone levels

Maternal saphenous vein serum and umbilical artery (i.e. fetal vessel) estradiol levels were reduced by approximately 95% (*P* < 0.01) in letrozole-treated baboons (Table 1). Maternal estradiol levels were returned to normal by letrozole plus estradiol administration. Although mean (± s.e.) umbilical artery estradiol levels in letrozole plus estradiol-treated baboons (0.08 ± 0.01 ng/mL) were higher (*P* < 0.01) than in animals treated only with letrozole (0.03 ± 0.00 ng/mL), levels were substantially lower than in untreated baboons, because of placental metabolism of maternally administered estradiol (Albrecht & Pepe 1990).

**Table 1:** Maternal serum estradiol levels, fetal baboon body weight, and offspring basal blood glucose and plasma insulin levels.

	Serum estradiol (ng/mL)		Fetus	Offspring	
Treatment	Maternal saphenous	Umbilical artery	Body weight (g)	Glucose (mg/dL)	Insulin (µIU/mL)
Untreated	3.12 ± 0.45^a^	0.71 ± 0.11^a^	875 ± 32	68 ± 5	4.2 ± 0.8^a^
Letrozole	0.23 ± 0.01^b^	0.03 ± 0.00^b^	802 ± 41	72 ± 2	8.2 ± 1.2^b^
Letrozole + estradiol	3.42 ± 0.17^a^	0.08 ± 0.01^c^	833 ± 29	70 ± 2	5.0 ± 0.7^a^

Values are expressed as the mean ± s.e. in fetuses and in 4–8-year-old adult offspring on day 165 of gestation delivered to maternal baboons untreated (*n* = 16) or treated on days 100–164 (term = 184 days) via maternal s.c. injection with letrozole (115 µg/kg body weight/day, *n* = 18) or with letrozole (115 µg/kg body weight/day) plus estradiol benzoate (25 µg/kg body weight/day on day 100 increasing to 115 µg/kg body weight/day on days 120–165, *n* = 15). Values with different letter superscripts are significantly different (*P* < 0.01, estradiol and *P* < 0.05 insulin) from one another.

Mean (± s.e.) basal plasma insulin levels were two-fold higher (*P* < 0.05) in offspring from letrozole-treated baboons (8.2 ± 1.2 µIU/ml) than untreated or letrozole plus estradiol-treated animals (Table 1). However, offspring basal blood glucose levels and fetal body weights were similar in untreated, letrozole-treated, and letrozole plus estradiol-treated baboons (Table 1).

### VEGF expression and capillary density in fetuses

**Figure 1:**
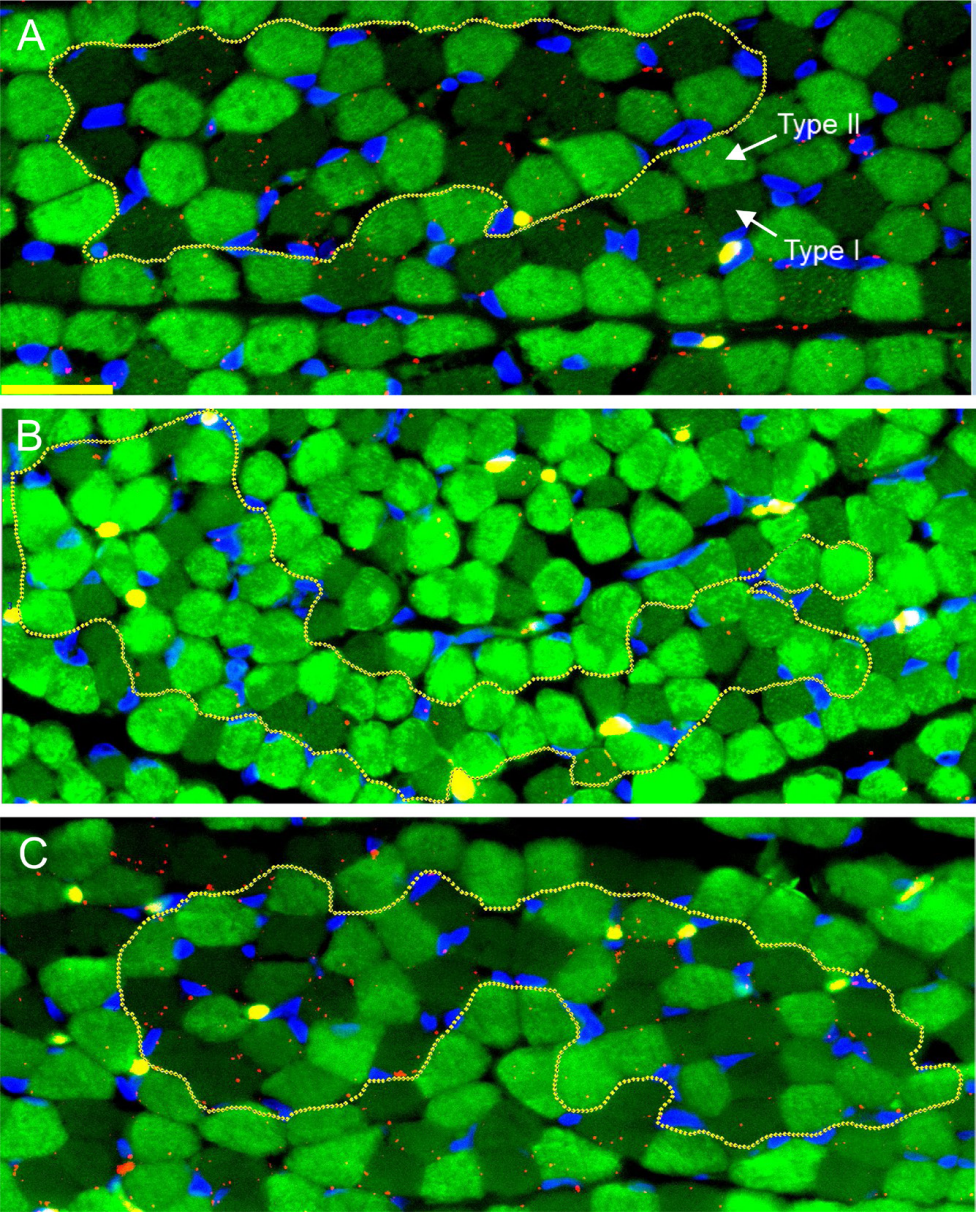
Photomicrographs illustrating the localization of small red VEGF immunofluorescent signals generated by proximity ligation assay between the skeletal muscle myofibers of fetuses on day 165 of gestation delivered to maternal baboons untreated (A), treated with letrozole (B), or treated with letrozole plus estradiol (C) as detailed in Table 1 footnote. Arrows label type II and type I myosin fast myofibers. White encircled areas represent randomly selected regions of interest of groups of muscle fibers used for proximity ligation assay image quantification. Yellow scale bar, 25 µm.

**Figure 2:**
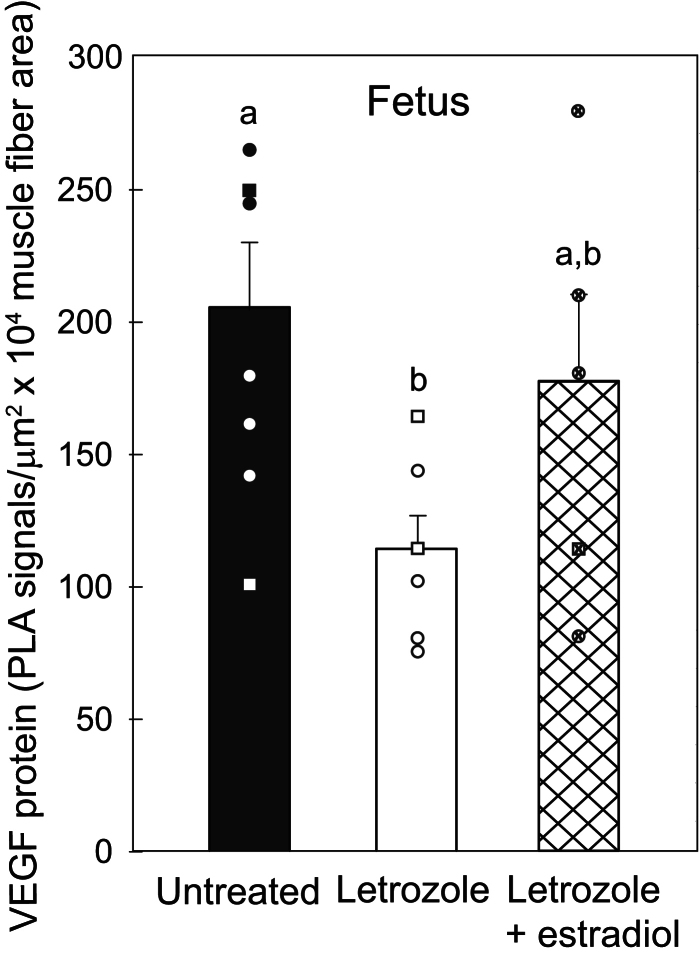
Mean ± s.e. (and individual data points) VEGF protein expression quantified by proximity ligation assay (A, number of VEGF immunofluorescent signals/µm^2^ × 10^4^ area of groups of skeletal muscle fibers) in skeletal muscle of fetuses on day 165 in baboons untreated (*n* = 7), treated with letrozole on days 100–164 (*n* = 6), or treated with letrozole plus estradiol (*n* = 5). Values of bars with different letter superscripts are different from each other (*P* < 0.05). Data points are designated as circles (females) and squares (males).

Figure 1 illustrates the localization of the small red VEGF immunofluorescent proximity ligation assay signals between the skeletal muscle myofibers of baboon fetuses. In contrast to the abundant expression of VEGF protein signals in fetal skeletal muscle of untreated baboons (Fig. 1A), VEGF signals appeared less numerous in letrozole-treated animals (Fig. 1B) and restored to normal by letrozole plus estradiol administration (Fig 1C). Thus, when quantified by MetaMorph image analysis, mean (± s.e.) VEGF protein expression (i.e. number of proximity ligation assay signals/µm^2^ × 10^4^ myofiber area) in fetuses of letrozole-treated baboons (113 ± 14) was 45% lower (*P* < 0.05) than in untreated animals (206 ± 24) and restored to normal with concomitant maternal letrozole plus estradiol treatment (177 ± 33, Fig. 2).

**Figure 3:**
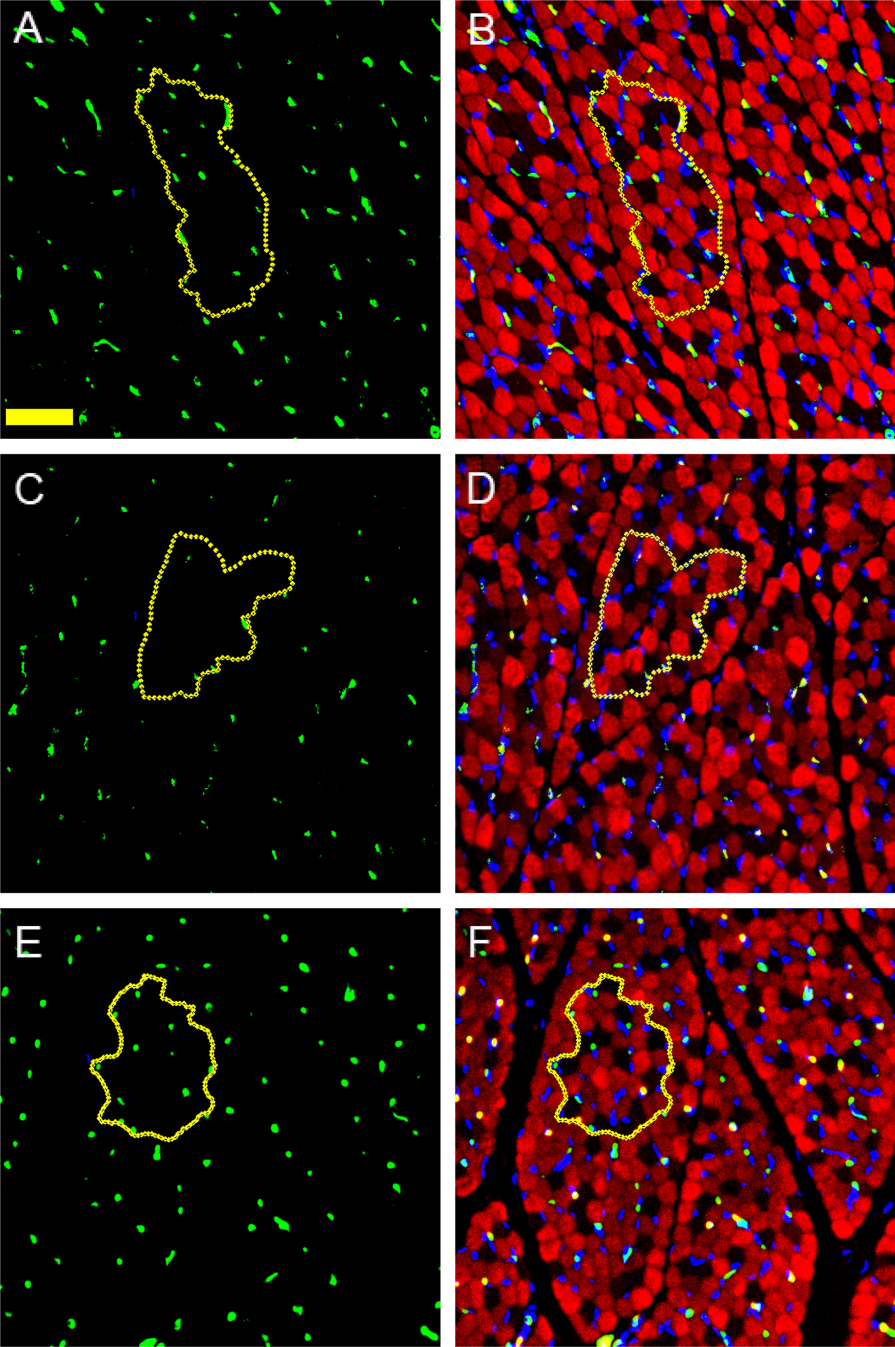
Photomicrographs illustrating localization of green von Willebrand factor-positive vascular endothelial cells between skeletal muscle myofibers of fetuses delivered on day 165 to maternal baboons untreated (A, B), treated with letrozole (C, D), or treated with letrozole plus estradiol (E, F). Green image channel (A, C, E); merged green, red, and blue image channels (B, D, F). Myosin fast myofibers (red, type II; black, type I); DAPI positive nuclei (blue). White encircled areas represent randomly selected regions of interest of groups of muscle fibers used for immunofluorescent image quantification. Yellow scale bar, 25 µm.

**Figure 4:**
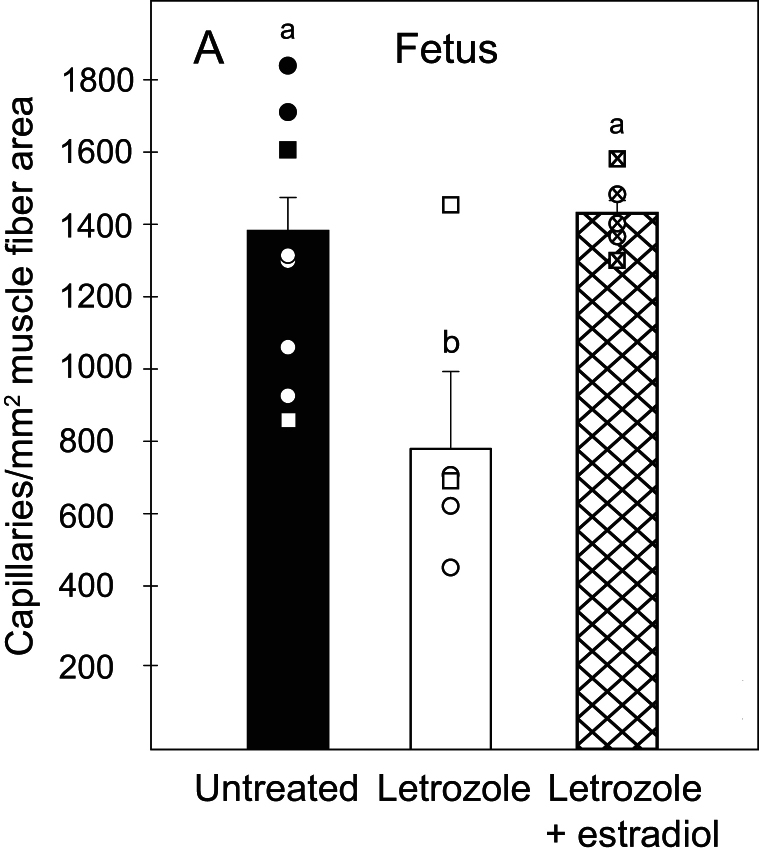
Mean ± s.e. (and individual data points) capillary density (number of capillaries/mm^2^ muscle fiber area) quantified by immunohistochemical image analysis in skeletal muscle of fetuses on day 165 in baboons untreated (*n* = 8), treated with letrozole on days 100–164 (*n* = 5), or treated with letrozole plus estradiol (*n* = 5). Values of bars with different letter superscripts are different from each other (*P* < 0.05). Data points are designated as circles (females) and squares (males).

Figure 3 illustrates the localization of green von Willebrand Factor positive fetal microvascular (i.e. capillary) endothelial cells between type II (red) and type I (black) myofibers as visualized within the green (A, C, E) and merged green, red, and blue (B, D, F) image channels. Since 3D microscopy has shown extensive embedding of capillaries within the sarcolemma of skeletal muscle fibers (Glancy *et al.* 2014), it is likely that the localization of von Willebrand positive microvessels reflected localization of capillaries within the sarcolemma of baboon skeletal muscle. The number of capillary endothelial cells appeared less numerous in the fetus of letrozole treated (C, D) than in untreated baboons (A, B) and restored to normal with letrozole and estradiol administration (E, F). Thus, as quantified by MetaMorph image analysis, the mean ± s.e . number of capillaries per mm^2^ myofiber area in fetuses of letrozole-treated baboons (783 ± 174) was 42% lower (*P* < 0.05) than in untreated animals (1343 ± 129) and returned to normal by letrozole plus estradiol treatment (1426 ± 49, Fig. 4).

**Figure 5:**
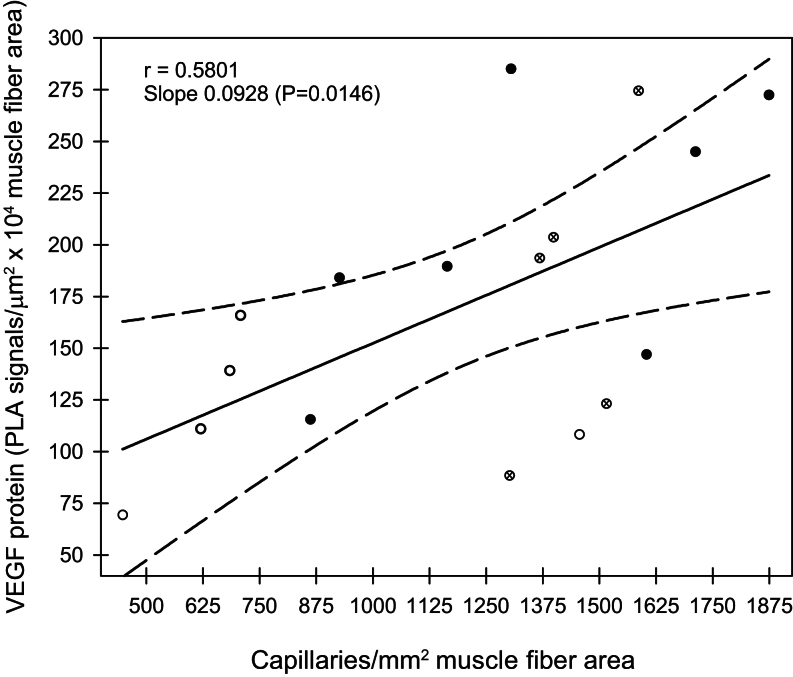
Linear regression, with 95% CIs (dashed lines), of skeletal muscle VEGF protein expression quantified by proximity ligation assay (PLA) and capillary density in fetuses obtained on day 165 of gestation from maternal baboons untreated (*n* = 7, ⚫), treated with letrozole (*n* = 5, ⚪) or treated with letrozole plus estradiol (*n* = 5, ⊗). Regression coefficient (*r* = 0.5801), slope = 0.0928 (*P* = 0.0146).

Linear regression analysis indicates that an increase in the expression of skeletal muscle microvessel VEGF protein was correlated (*r* = 0.5801, slope = 0.0928, *P* = 0.01) with elevation in the number of microvessel capillaries per myofiber area in fetuses from baboons untreated, treated with letrozole, or treated with letrozole plus estradiol (Fig. 5).

### Brachial artery dilation and mean arterial blood pressure

**Figure 6:**
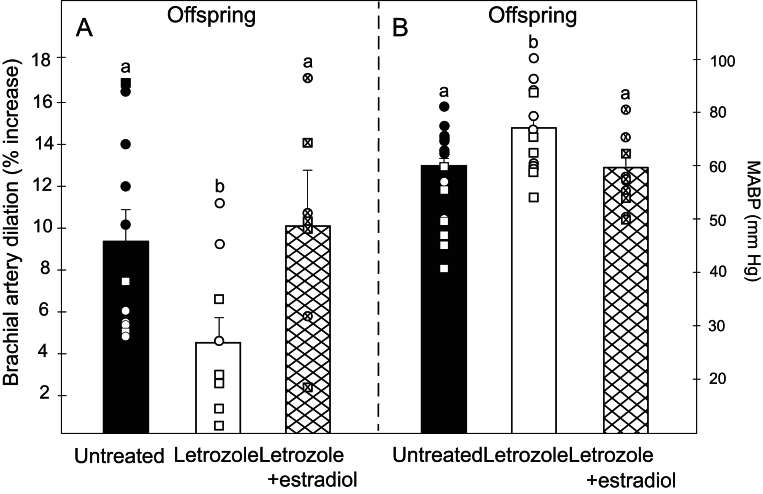
Mean ± s.e. (and individual data points) percent increase (versus basal) in brachial artery diameter after acetylcholine administration (A) and basal mean arterial blood pressure (MABP, mm Hg) in postpubertal offspring delivered to maternal baboons untreated (*n* = 11–15), treated with letrozole (*n* = 8–12), or treated with letrozole plus estradiol (*n* = 7–10). Values of bars with different letter superscripts are different from each other (panel A, *P* = 0.02; panel B, *P* = 0.01). Data points are designated as circles (females) and squares (males).

Mean (± s.e .) acetylcholine-induced brachial artery dilation in letrozole-treated baboon offspring (4.5 ± 1.3%) was 52% lower (*P* = 0.02) than in untreated animals (9.4 ± 1.4%) and restored by maternal administration of letrozole plus estradiol (10.1% ± 2.2%, Fig. 6A).

Basal mean arterial blood pressure was 23% higher (*P* = 0.01) in offspring of letrozole-treated baboon offspring (73.9 ± 4.0 mm Hg) than in untreated animals (59.9 ± 3.1 mm Hg) and returned to normal by administration of letrozole plus estradiol (59.8 ± 3.9 mm Hg, Fig. 6B).

The relatively low number of female and male fetuses and offspring did not provide sufficient statistical power to determine whether there was a statistically significant sex difference with respect to the measurement of skeletal muscle VEGF levels, microvessel density, or vascular reactivity.

### Glucose tolerance test

**Figure 7:**
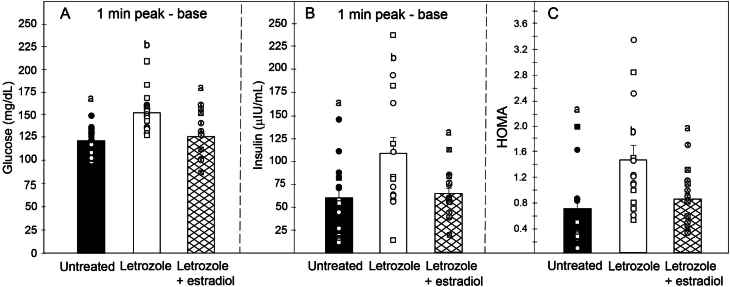
Mean ± s.e. (and individual data points) blood glucose levels (A, 1 min peak-base), plasma insulin levels (B, 1 min peak-base), and homeostasis model of insulin resistance (C, i.e. HOMA, basal blood glucose level × basal plasma insulin level / 405) in postpubertal offspring delivered at term to maternal baboons untreated (*n* = 14–16), treated with letrozole (*n* = 14–18), or treated with letrozole plus estradiol (*n* = 14–15). Values of bars with different superscripts are different from each other (panel A, *P* < 0.001; panel B, *P* < 0.05; panel C, *P* < 0.01). Data points are designated as circles (females) and squares (males). Individual values for glucose (1 min peak-base) in offspring untreated: 102, 127, 99, 115 (current study) and 101, 123, 101, 125, 149, 136, 131, 107, 125, 135, 131, 99 (Albrecht *et al.* 2022); letrozole: 168, 134, 129, 160, 155, 134, 162, 211, 184, 158 (current study) and 141, 149, 150, 134, 154, 135, 159, 127 (Albrecht *et al.* 2022); letrozole plus estradiol: 117, 128, 87, 131, 101, 156, 110 (current study) and 112, 135, 128, 127, 100, 141, 161, 151 (Albrecht *et al.* 2022). Individual values for insulin (1 min peak-base) in offspring untreated: 149, 72, 83, 58 (current) and 14, 53, 111, 16, 44, 71, 27, 16, 12, 88, (Albrecht *et al.* 2022); letrozole: 119, 83, 58, 184, 237, 81, 164 (current) and 14, 56, 111, 196, 62, 61, 71 (Albrecht *et al.* 2022); letrozole plus estradiol: 44, 78, 86, 119, 83, 58, 58 (current) and 21, 41, 58, 40, 61, 61, 76 (Albrecht *et al.* 2022). Individual values for the HOMA in offspring untreated: 0.87, 0.89, 0.81, 2.02 (current) and 0.50, 0.36. 0.10, 1.62. 0.29, 0.33, 0.32, 0.26, 0.25. 0.88 (Albrecht *et al.* 2022); letrozole: 0.56, 2.89, 1.03, 0.84, 1.28, 0.78, 1.23 (current) and 0.60, 1.14, 1.11, 1.48. 2.55. 1.53. 3.37 (Albrecht *et al.* 2022); letrozole plus estradiol: 0.87, 0.45, 1.33, 0.36, 1.10, 0.50, 0.93 (current) and 0.36; 1.79; 1.17; 1.07; 0.63; 0.47, 0.88 (Albrecht *et al.* 2022).

Figure 7 includes the glucose tolerance test data obtained in a new cohort of baboons and that obtained in our previous study (Albrecht *et al.* 2022). The mean (± s.e.) 1 min peak-basal levels of fasting glucose and insulin after the bolus injection of dextrose in offspring delivered to letrozole-treated baboons (152 ± 5 mg/dL and 107 ± 17 µIU/mL, respectively) were 28% and 84% higher (*P* < 0.001 and *P* < 0.05, respectively) than in offspring of untreated animals (119 ± 4 mg/dL and 58 ± 11 µIU/mL, respectively) and returned to normal by administration of letrozole plus estradiol (125 ± 5 mg/dL and 63 ± 6 µIU/mL, respectively, Fig. 7A and B). Consequently, the homeostasis model of insulin resistance was 114% higher (*P* < 0.01) with letrozole treatment (1.46 ± 0.23) compared with that with no treatment (0.68 ± 0.15) and returned to normal with the administration of letrozole and estradiol (0.85 ± 0.11. Fig. 7C). Although the number of female and male offspring in which a glucose tolerance test was performed provided sufficient power for statistical analysis, there was no statistically significant effect of sex of the offspring on the levels of glucose (*P* = 0.45), insulin (*P* = 0.84), or the HOMA (*P* = 0.34).

## Discussion

The present study shows that expression of VEGF was decreased and correlated with a comparable reduction in capillary density, within the skeletal muscle of fetuses delivered from baboons in which maternal and fetal serum estradiol levels were suppressed during the second half of pregnancy by the administration of letrozole. The decrease in fetal VEGF expression and capillary density was prevented by maternal administration of letrozole plus estradiol. These findings indicate that estrogen during primate pregnancy has a pivotal role in simultaneously promoting systemic skeletal muscle VEGF expression and microvessel development within the fetus. Importantly, the reduction in fetal VEGF expression and microvascular development in estrogen-deprived baboons was subsequently manifest as a comparable decrease in offspring systemic microvessel density as shown previously (Albrecht *et al.* 2022), and impaired systemic vascular response to acetylcholine, increase in mean arterial blood pressure, as well as insulin resistance and glucose intolerance in offspring of the present study. Each of these perturbations in vascular and metabolic function in estrogen-deprived offspring was prevented by supplementing maternal estrogen during letrozole administration. Therefore, the collective results of the recent and current studies are highly significant in showing that estrogen, a major component of the hormonal milieu of pregnancy, upregulates VEGF expression and microvascular development within the nonhuman primate fetus, as an essential programming event for the ontogenesis of vascular function and insulin action within target cells after birth in the progeny. Although we propose that VEGF mediates the stimulatory action of estrogen on fetal microvascularization, additional experimental paradigms are required to definitively demonstrate causality of the proposed mediatory action of VEGF.

Although we had shown previously that the ratio of the number of capillaries to the number of myofibers was reduced in estrogen-deprived baboon fetuses (Albrecht *et al.* 2022), the size of the fetal myofibers was also decreased (Kim *et al.* 2022). The present study shows that capillarization of the area of fetal myofibers was comparably decreased by letrozole administration and restored by maternal letrozole plus estradiol treatment. We propose, therefore, that the overall responsivity of skeletal muscle, which accounts for over 80% of insulin-directed glucose uptake and metabolism, to insulin would be compromised in the offspring by *in utero* estrogen deprivation.

The present study also demonstrated that arterial vasodilation was impaired during acetylcholine infusion of offspring born to estrogen-deficient baboons. This finding, along with the decrease in arterial vasodilation after FMD-induced hyperemia shown previously (Albrecht *et al.* 2022), indicates that collective nitric oxide- and prostaglandin-modulated arterial reactivity is compromised after birth by disrupting the estrogen-enriched milieu of pregnancy.

The appearance of insulin resistance in offspring delivered to estrogen-suppressed baboons did not differ with respect to sex of the offspring, as shown in the present study, or the onset of puberty, as shown previously (Maniu *et al.* 2016, Pepe *et al.* 2016). Thus, the insulin resistance exhibited in prepubertal offspring born to letrozole-treated baboons (Maniu *et al.* 2016) was sustained in postpubertal animals despite the elevation in estradiol or testosterone that accompanies puberty in females and males. However, estrogen confers beneficial and androgen deleterious effects on the incidence of diabetes in women and men (Gale & Gillespie 2001, Margolis *et al.* 2004, Mauvais-Jarvis *et al.* 2013, Navarro *et al.* 2015). The retainment of insulin insensitivity in postpubertal offspring delivered to estrogen-deprived baboons highlights the importance of estrogen *in utero* in programming metabolic homeostasis after birth.

The stimulatory effect of estrogen on fetal baboon skeletal muscle VEGF expression and microvessel development is consistent with the well-established action of VEGF on vascularization. Thus, VEGF promotes skeletal muscle capillary proliferation and function (Ishii *et al.* 2002, Olsson *et al.* 2006, Wagner *et al.* 2011, Hellsten & Hoier 2014, Olfert *et al.* 2010, Bonner *et al.* 2013) in many tissues of the adult, and deletion of VEGF in transgenic mice results in capillary regression and insulin resistance (Bonner *et al.* 2013). This points to the potential of VEGF, as well as estradiol, as therapeutic modalities to prevent or reverse the development of fetal microvessel rarefaction and offspring metabolic dysfunction that arise from disruption of the hormonal milieu of pregnancy.

The onset of insulin resistance elicited in offspring delivered to estrogen-deprived baboons, as shown previously (Albrecht *et al.* 2022), as well as in the current study, has substantial translational significance. Thus, disruption of the levels or action of estrogen during the second half of human pregnancy impairs microvessel development and insulin sensitivity after birth. For example, preterm birth, which occurs in approximately 10% of all pregnancies, curtails exposure of the fetus to the progressive increase in estrogen during advancing stages of pregnancy and is associated with microvessel rarefaction and insulin resistance in childhood and adulthood (Hofman *et al.* 2004, Bonamy *et al.* 2007, Darendeliler *et al.* 2008, Paz *et al.* 2017). Moreover, aromatase gene mutation and elevated levels of endocrine disruptors that bind to and interfere with estrogen receptor action during pregnancy lead to T2DM in offspring (Fukami *et al.* 2009, Czajka-Oraniec & Simpson 2010, Schönfelder *et al.* 2002, Alonso-Magdalena *et al.* 2010, Chevalier & Fénichel 2015). However, human prematurity is associated with low birth weight and significant complications of renal, hepatic, and metabolic function (Gluckman *et al.* 2008, Thompson & Regnault 2011), making it difficult to definitely establish the impact of estrogen depletion. An advantage of the present experimental paradigm to study the relationship between *in utero* estrogen and offspring metabolic function is the selective decrease in the levels of estrogen in the absence of changes in maternal glucose tolerance, umbilical blood flow, placental villous vascularization, birth weight, and analytes reflecting liver and kidney function (Robb *et al.* 2007, Aberdeen *et al.* 2010, Maniu *et al.* 2016).

Although umbilical artery (i.e. fetal vessel) estradiol levels near term were increased to 80 pg/mL after letrozole plus estradiol administration, this value was substantially lower than in untreated baboons. However, the levels of estrogen produced during normal human and nonhuman primate pregnancy are considerably higher than required to promote the estrogen-dependent physiological events of pregnancy (Albrecht and Pepe 1990). Moreover, the dissociation constants for estrogen receptor (ER)α and ERβ are 50 and 90 pM, or 13 and 24 pg/mL, respectively (Kuiper *et al.* 1998, Lin *et al.* 2013), which approximate the serum estradiol level achieved in the baboon fetus by maternal letrozole plus estradiol administration. In addition, serum estradiol levels of 50–80 pg/mL increase endometrial thickness during the proliferative phase of the human menstrual cycle (Dighe *et al.* 2005, Verdonk *et al.* 2019). Therefore, we suggest that the restoration of baboon fetal and offspring vascular development by letrozole plus estradiol treatment reflects an estrogen-specific action.

In summary, the present study builds on our previous study to show that estrogen promotes skeletal muscle VEGF expression and microvascular development in the fetus, and consequently, the onset of systemic vascular function as a requirement for insulin sensitivity/glucose homeostasis after birth in the offspring.

## Declaration of interest

The authors declare that there is no conflict of interest that could be perceived as prejudicing the impartiality of the study reported.

## Funding

This work was supported by the National Institutes of Health Researchhttp://dx.doi.org/10.13039/100005622 Grant R01 DK 120513.

## Data availability

Some or all data generated or analyzed during this study are included in this published article or in the data repositories listed in the references.
